# Provider-Level and Other Health Systems Factors Influencing Engagement in HIV Care: A Qualitative Study of a Vulnerable Population

**DOI:** 10.1371/journal.pone.0158759

**Published:** 2016-07-18

**Authors:** Yukyan Lam, Ryan Westergaard, Gregory Kirk, Azal Ahmadi, Andrew Genz, Jeanne Keruly, Heidi Hutton, Pamela J. Surkan

**Affiliations:** 1 Johns Hopkins Bloomberg School of Public Health, Baltimore, Maryland, United States of America; 2 University of Wisconsin School of Medicine and Public Health, Madison, Wisconsin, United States of America; 3 Johns Hopkins School of Medicine, Baltimore, Maryland, United States of America; International AIDS Vaccine Initiative, UNITED STATES

## Abstract

Despite the existence of highly active antiretroviral therapy, HIV/AIDS morbidity and mortality continue to be public health burdens in the United States due to difficulties in engaging people living with HIV/AIDS (PLWHA) in continuous, effective care. In comparison to studies investigating patient-level characteristics associated with starting and remaining in care, there is relatively little research on how structural factors, such as those pertaining to healthcare providers and the infrastructure for delivery of health services, influence patients’ engagement in HIV care. Our study, based in the city of Baltimore, Maryland, uses qualitative research methods with a population of predominantly African American PLWHA who have a history of drug abuse, to examine facilitators and barriers regarding adherence to antiretroviral therapy (ART) and HIV care appointment attendance. Data collection involved conducting one-on-one, in-depth interviews with 31 study participants, and data analysis entailed thematic coding of interview transcripts and writing analytic memos to develop ideas and concepts. Among other findings, factors described as influential by our study participants related to appointment reminders and scheduling, the attitudes and communication styles of HIV clinicians, and the disposition and availability of other healthcare workers on the care “team.” Thus, improving quality of HIV care and means of delivering it may help mitigate the numerous points in the continuum of HIV care when a patient may disengage.

## Introduction

In the United States, HIV/AIDS mortality is 2.2 per 100,000 residents [1]. In Baltimore City, Maryland, the rate is 21.1 per 100,000 residents [2]. Comparing the figures for different racial categories in Baltimore reveals a dramatic disparity: 6.3/100,000 in White non-Hispanics versus 30.5/100,000 for Black non-Hispanics. Black men and those with less than or only a high school education are the demographic groups with the highest rates of HIV/AIDS mortality [2].

HIV infection is a chronic condition that can be successfully managed with antiretroviral therapy (ART), which suppresses viral replication and halts immune system deterioration. ART involves taking medication on a daily basis, which can prevent progression to AIDS and enable persons infected with the virus to have nearly normal life expectancies [3–6]. Optimal HIV care is characterized by a continuum of care—early diagnosis of infection, prompt linkage to regular care, appropriate initiation of ART, high levels of medication adherence, and retention in care over the life course [7–9]. Without a vaccine or cure, reducing the burden of HIV/AIDS in places such as Baltimore City will require identifying, understanding and addressing the reasons why persons living with HIV/AIDS (PLWHA) do not have effective, continuous care.

Structural factors have been shown to be important for engagement in HIV care amongst US-based PLWHA [10–12] and highlighted in models, including socioecological frameworks delineating the interplay of intrapersonal, interpersonal, community, health system, and health policy factors that influence engagement in care [13]. Much of the prior research on the factors related to initiation and retention in care has been quantitative in nature [11, 14–17]. Relatively few studies have shown how specific structural barriers and facilitators operate from the patient’s perspective or how these factors interact. This is particularly true for factors related to the systems of support and types of communication used by healthcare providers. For example, a systematic review of barriers to ART adherence found that patient satisfaction with patient-provider relationships and the healthcare received was inconsistently associated with adherence [14]. A recent, Baltimore-based observational study found that some aspects of the patient-provider relationship were significantly associated with improved appointment attendance, however the study did not reveal how these factors were associated or provide insight into how the personal circumstances in patients’ lives played a role (e.g., poverty, substance abuse) [18]. Qualitative research that examines, in-depth, how the interactions among vulnerable patients, providers, and systems for administering healthcare can be improved, can aid in interpreting these quantitative study findings.

The aim of our study was to identify and elucidate higher-level facilitators and barriers for engaging in HIV care, specifically factors related to health systems. We understand health systems factors to include provider-level characteristics and the patient-provider relationship, as well as other features of the infrastructure in place for administering care. Using qualitative methods, we examined in detail both ART adherence and clinic appointment attendance among a high-risk population of Baltimore City-based, predominantly African American PLWHA who have a history of drug abuse.

## Methods

This qualitative analysis was conducted within the context of a larger study, entitled “Technology-Enhanced Peer Navigation to Improve Intravenous Drug Users’ Engagement in HIV Care: The mPeer2Peer Study” (hereinafter, “mPeer2Peer”). mPeer2Peer was a pilot prospective, randomized trial comparing the effectiveness of a nine-month multi-component, mobile phone-based intervention to standard medical case management for IDUs re-engaging in HIV care. The present qualitative investigation uses information, independent from the phone-based intervention, obtained from individuals in mPeer2Peer’s control and intervention arms after they were recruited into mPeer2Peer. To provide context on where and how participants were recruited, we briefly describe mPeer2Peer below.

### Study setting and population

mPeer2Peer was conducted in Baltimore, Maryland. The target population was HIV-infected adults with a history of past or current substance abuse disorder, who either had never started HIV clinical care or antiretroviral therapy (ART) or had ever experienced a gap in their treatment (appointments or ART). The inclusion criteria were a minimum of 18 years of age, HIV-infected with viral load greater than 1000 copies/mL, no clinic visits with an HIV care provider in the preceding six months, and willing to attend at least one HIV care visit at the Johns Hopkins Moore Clinic. A prior diagnosis of substance use disorder was an inclusion criterion in the original mPeer2Peer protocol, but the requirement was removed mid-study because of difficulty meeting recruitment goals. Exclusion criteria were any medical or psychiatric conditions that would interfere with the participant’s ability to comply with study procedures (e.g., eyesight conditions that would make it difficult to read the smartphone screen) and concurrent participation in other studies focusing on HIV care retention. Active drug use was not an exclusion criterion.

### Recruitment into mPeer2Peer

mPeer2Peer study enrollment occurred between September 2013 and November 2014. Individuals were screened for eligibility if they were active participants in the AIDS Linked to the IntraVenous Experience (ALIVE) study, an on-going cohort study based in Baltimore [19]. Additional recruitment occurred on-site at the Moore Clinic, where providers or case managers identified patients known to be HIV-positive who were not engaged in regular care.

After providing written consent, participants attended a baseline visit, during which they were randomized to either the control or intervention arm. Participants in the qualitative study were recruited from both arms of the mPeer2Peer intervention. All participants then completed a baseline demographic form, and intervention arm participants completed an additional barriers and facilitators questionnaire. Qualitative interviews, described below, were conducted either during the same baseline visit or at a separate visit. For a few participants (those recruited in the initial months of mPeer2Peer), their qualitative interviews occurred after they had already started receiving the mPeer2Peer intervention; however, the focus of the interviews and this qualitative study was not on the intervention, but rather participants’ long-term history of HIV care engagement (challenges faced, etc.) prior to being recruited into mPeer2Peer.

The study was approved by the Johns Hopkins Bloomberg School of Public Health Institutional Review Board (IRB). The IRB approved the consent forms used to obtain written consent from the participants. In addition, this study was registered in September 2013 at ClinicalTrials.gov (NCT01941108).

### Qualitative data collection

Between February and November 2014, we conducted one-on-one, in-depth interviews with 15 control arm participants and 16 intervention arm participants enrolled in mPeer2Peer. Because of timing and participant availability, our qualitative study drew on information obtained from 31 of the total 39 participants enrolled in mPeer2Peer. The interviewers were three doctoral students at the Johns Hopkins Bloomberg School of Public Health with graduate-level training in qualitative methods. Their approaches were standardized through training and the use of an interview guide. Interviews were semi-structured, based on the following topics outlined in the guide: experiences with starting and stopping HIV care (both appointments and medication), reasons for starting and stopping HIV care, circumstances that made it difficult for them to adhere to ART or attend appointments, and factors that made or would make it easier for them to adhere to ART or attend appointments. To facilitate probing, interviewers also had access to the demographic questionnaires and, for the intervention arm participants, barriers/facilitators questionnaires completed at the baseline visit. All interviews were digitally recorded. Interviews were on average 36 minutes (ranging from 14 to 104 minutes). Participants were reimbursed for travel expenses to the interview location, but were not otherwise paid for participating in the qualitative interviews.

### Qualitative data analysis

All interviews were transcribed, and transcripts were imported into MaxQDA, a software program for managing and analyzing qualitative data (MaxQDA: Release 11. Statistical Software. Berlin, Germany: VERBI Software). Qualitative data analysis was based on the method of thematic analysis, which aims to discover themes, as defined as patterns of responses or meaning within the data, through a process of coding, analysis, thematic mapping, and theme naming and definition [20]. Our analysis used both deductive and inductive approaches, as follows: A preliminary codebook was developed largely based on the barriers and facilitators questionnaire. Thereafter, two of the authors independently coded two full transcripts and noted areas where the codebook should be modified to account for unexpected themes. They collaboratively revised the codebook and used it as a basis for coding all of the transcripts. Throughout the coding process, the two coders met to clarify code definitions and decide on revisions to the codebook based on emerging themes.

The final codebook had four major domains: (i) demographic characteristics, (ii) HIV diagnosis and treatment history, (iii) barriers to care, and (iv) facilitators of care. Each of the ‘barriers’ and ‘facilitators’ domains had a ‘health systems’ category, the findings of which are the focus of this paper. When possible, our analysis also distinguished between appointment attendance and medication adherence, as well as between actual facilitators and hypothetical facilitators (i.e., when a participant said that something *actually* helped them versus *might* help them engage in care). After coding, we generated code reports and analyzed the coded segments further by organizing them into themes. We complemented this coding process with analytic memo-writing, which developed ideas and concepts further. Finally, we refined the themes and re-read the transcripts to verify their significance.

## Results

Our sample consisted of 12 women and 19 men (mean age 49.8, SD = 7.9 years). Approximately 90% of the participants self-identified as non-Hispanic Black. About 80% had a history of cocaine or heroin use. Three participants were currently married or in a common law marriage, three were widowed, six were divorced, and the remaining 19 had never been married. Nearly one-third of the participants had made less than $2500 in legal income during the previous year, and about four-fifths had made less than $10,000. All of the individuals in our sample had had some kind of HIV care previously, i.e., they had taken medication and/or attended a medical appointment for HIV at least once. Among the 26 participants who recalled and reported the year when they first tested positive for HIV, the years ranged from 1984 to 2010, with most (17 out of 26) reporting a year between 1989 and 1998.

Our data analysis of the “health systems” sub-categories revealed themes that could be grouped into three major areas. Starting with the most salient (i.e., mentioned the most often and foremost), the topics were: (i) appointment and medication logistics, (ii) clinicians, and (iii) other healthcare workers.

### Appointment and medication logistics

One important determinant of successful engagement in care was the appointment and reminder system. Nearly all participants reported that being reminded of appointments by letter, phone call, or text message was crucial in ensuring that they kept their appointments:

“They send you flyers, reminders of all your appointments. They mail them to your house. So, even if you forget your appointment, it’s still coming to your door unless you don’t pick up mail and read.”

(Female, 43 years old)

When probed about why they might have missed appointments in the past, some participants mentioned that they had forgotten because of the time elapsed between appointments. As one 52-year old male participant explained, “I just can't go a long ways, remember a long ways like that, so that's why they call me or they get that letter, and then I do it.”

Another important facilitator was getting an appointment that did not conflict with their work schedule, as several participants implicitly or explicitly stated that they would choose getting paid over going to see the doctor. In this regard, one 55-year old male described the decision as an obvious one:

“If they set [the appointment] on a day that I have to work, the choice I have is I'm coming to my appointment or is I'm going to work? I'm going to work, because I got to eat. Seeing them ain't going to feed me.”

(Male, 55 years old)

Another participant explained that inconvenient appointment times served as a deterrent, but was not an absolute barrier, as it varied occasion by occasion:

“[My doctor’s] schedule is usually full and that’s the time they’ve been giving me. So I deal with it, and then at times I just say, ‘forget it.’ It’s too late, and I just don’t go.”

(Female, 46 years old)

In most cases, participants perceived getting the appointment itself—which involved making a phone call or scheduling the next appointment before leaving the current appointment—as a relatively easy process. However, it could be a challenge getting an appointment soon. A few participants mentioned that it might require waiting months before the next available appointment slot. This was attributed to there being too many patients per doctor and/or the doctor only coming in one day per week. As one man explained:

“They got so many clients and patients, the appointment is gonna be a couple months…. So I had inquired about seeing [my doctor] more often, and that was the story they gave me of why the appointments are so far apart like that, because he’s only there once a week and he got to see [them] all.”

(Male, 49 years old)

The same participant explained that, as a result of not being able to get timely appointments, the time elapsed made it easier to forget future appointments. Another reported that long periods between appointments made it difficult to get advice on a more urgent medical question:

“If I find myself missing [doses of the medication for a] couple days, I would just stop completely…. At times like that, I’d be wanting to talk to my doctor, but then I can’t because I got to call them, and he gonna schedule an appointment two months away.”

(Male, 49 years old)

However, another participant emphasized the opposite view that his appointments were *too* frequent—only a few weeks apart—giving rise to the feeling that the visits wasted his time and were designed to make money for the healthcare providers:

“If I don't feel bad and I feel okay, I don't need the appointments the way she have them…. Sometimes when she want to she'll make them every month. Come on, now. Why you going to go from every three months down to a month or from a month down to two weeks? No, you're not going to do that to me….You making money.”

(Male, 55 years old)

Regarding the experience of attending the appointment, many study participants cited long waiting times when they arrived for the appointment as a problem; however this was by no means a universal complaint. Nor was it explicit that this might deter patients from attending appointments in the future. Nevertheless, long waiting times could make transportation difficult, interfere with one’s job, or make participants feel frustrated or like their time was being wasted:

“You got a thousand more patients out there, and they're out there getting rotted because of the fact of you taking up their time just like you taking up my time…. It's unnecessary. That's very unnecessary. And I don't call that being professional. See what I'm saying? Because I'll be sitting out there in the waiting room. I watch the doctors that come get their patient…. Hey, I'm still sitting here waiting to go in there to see her.”

(Male, 55 years old)

An extended waiting period could even interfere with picking up medications, as one 49-year old male participant told us: “I went yesterday, my appointment was yesterday. You see, I’m going back today to get my meds. I was not gonna sit there for another 45 minutes waiting. ‘Really? I’ve been here for four hours already. I’m not waiting.’” The participant explained further that accounting for the waiting times and the various things a patient might need to take care of during a single visit, the time spent could be quite substantial:

“Your appointment is at ten o’ clock, you might get out of there at 4:30, 5:00. You gotta go see your doctor, you wait to see your doctor. You know, your appointment might be ten o’ clock, you might not see your doctor until 10:45. Then you're in there with your doctor until twelve o’ clock. Then you gotta go down to blood work, you might be in there for another thirty minutes. Then if you gotta go see social work, you're there for another two hours. Meanwhile, you come outside you got a ticket because parking is only for two hours, you know, it's just crazy.”

(Male, 49 years old)

On the other hand, the same participant recognized that spending the whole day taking care of multiple things in one visit might be preferable to coming in for shorter visits every couple of weeks. With his previous appointment schedule, the burden of coming in once or twice a month, especially when combined with his work obligations, was “becoming just too much.”

To a somewhat lesser extent, logistics for acquiring medication refills also emerged as a barrier or facilitator for continuing care. Several participants described a system where they would call when they were close to running out of their current medication, and then the refill would be delivered to their home. This was seen as facilitating adherence. In fact, another participant specifically mentioned that picking up medications from the pharmacy in person was difficult given his academic schedule and the hours that the pharmacy was open.

While several participants mentioned that it was easy enough to know when to call to place a refill order, one person mentioned a need for reminders about when to call his insurance company to get the refills, given that he had to keep track of several types of medication. In one case, a participant’s physician played a direct role in calling to make sure that the patient’s medication was in stock at the pharmacy whenever he needed to go and pick up a refill, and this support was perceived as helpful:

“She makes sure my medicine is up to date and it's always in the pharmacy when I go there. She calls up regularly and make sure that it's there if I need it and I just go down to the Moore Clinic pharmacy and pick it up and take my medicine regularly so I never runs out. And it's a good thing that they have that kind of program 'cause it helps people out quite a lot.”

(Male, 53 years old)

### Clinicians

Factors related to clinicians themselves also emerged as important determinants of engagement in HIV care. The clinicians referred to in the qualitative interviews included both physicians outside and within the Moore Clinic (because interviewers probed about participants’ history of engagement in HIV care prior to being recruited into mPeer2Peer, as well as their perceptions of clinicians in general). Participants discussed their physicians’ communication styles, their disposition and attitude when interacting with patients, and their ability to balance explaining care-related matters sufficiently to patients against seeming repetitive or pedantic.

In general, participants had very positive comments about clinicians who followed up with them and “ke[pt] a tight relationship.” This included both good communication face-to-face during the appointment, as well as follow-up communication by phone or texting. As one 46-year old woman explained: “I love my doctor…. She’s there whether I’m visiting, [have] an appointment, or if I just need to call and talk to her, she’s there.”

Good communication with clinicians was a facilitator that motivated patients to make appointments, as well as stay on medications. To the extent that they felt invested in or cared about, patients worked harder to continue their treatment. A 61-year old male who had just re-engaged in HIV care described: “I’m in the Moore Clinic now. They gonna help me a lot. You know, they tell me to keep trying. ‘Don’t give up.’ ‘Don’t stay away from them too long.’ ‘Take your medicine every day.’ And that’s what I’m going to do.” Another participant who had a history of depression and substance abuse similarly described his current physician:

“He’s been the best doctor I’ve ever had, and being that I know I have a good rapport and relationship with him and that he sees the best in me in that state of mind, that’s what also made me, you know, wanna do the right thing.”

(Male, 36 years old)

Topics discussed could range from treatment regimens and side effects to additional resources, like social workers and services. Describing how his current doctor differed from his previous ones, the same participant stated:

“They follow through. If I leave a voicemail about a question, immediately, they call back before the end of the day and answer that question. Whether it be I'm not understanding why this medicine does this side effect, or why should I take this four times a day, what's going on… Or additional resources, you know, far as social work and stuff like that, they can access me to.”

In contrast, a few participants did not like being told what to do or being patronized by doctors. The importance of balancing a caring or firm attitude against seeming patronizing was reflected in one participant’s complaint about his physician, as follows:

“Sometimes she irks me, because she try to act like she’s my mother—but that’s all part of just caring, because she cares, and I can understand that. But sometimes she takes it too far…. Don’t tell me what to do. Ask me what to do…. When you try to tell me what to do, then I lose it.”

(Male 55, years old)

A few participants also reported feeling like their doctors did not listen to them or know them, and thus were unable to address their needs. One 47-year-old male who had last seen a physician for HIV care two years ago succinctly stated: “He just didn’t know me…so I just stopped going.” Another participant complained about her HIV doctor’s general attitude and failure to listen to her:

“I try to explain to him: ‘I know my body. I know I’ve been on drugs, but I know my body more than you do because you have to run tests.’ … And he’s a good doctor, but he don’t listen to me…. It’s like he’s not really worrying about what I’m telling him that’s wrong with me or how I feel. Like he’s not interested.

(Female, 50 years old)

Poor communication or ‘weak’ patient-provider relationships could also take the form of clinicians failing to impart sufficient information to participants. Referring to care under his previous physicians as a “horrible” experience, one participant recounted:

“I was not following through or not really asking questions, getting knowledge of what I was dealing with because I felt like it was their responsibility [to tell me], not mine [to ask]. So I was like, ‘you’re not telling me what I need to know.’ ‘Oh, just take these pills and be done with it.’ As I look back, I should’ve asked questions…. I didn’t know what was going on. I just took the pills and that was it.”

[Male, 36 years old]

Participants viewed their relationship with their doctors as affecting their appointment attendance, as well as whether they started or adhered to medication. For example, having sufficient information about how to take medications could help someone know what to do if he/she missed a dose, or having information about potential side effects could make the effects easier to manage. At the very least, having thorough communication about their treatment regimens could help patients feel more engaged in their care. For example, when asked to name some factors that facilitated staying on medication, one 52-year old man responded, “[My doctor] talking to me. That’s it. She just tell[s] me about it.” When asked whether any aspect about going to doctors’ appointments was helpful for staying in care, another participant replied:

“Well, to talk to my doctor, yes, [it is helpful]. And that’s because she gives me a lot of insight on the different medications, on how long a medication will last me if I stay on it and take it the way I’m supposed to. So me and my doctor talk about a lot. We go into a lot as far as care.”

[Male, 26 years old]

For a couple participants in our study, finding a clinician with whom they could develop good rapport was complicated by high clinician turnover, which prevented retaining/building on relationships, and not knowing where to look for a good provider. Illustrating the high value placed on a strong patient-doctor relationship, one participant even preferred to keep the same clinician even though the clinician’s schedule led to appointment times that would conflict with his work obligations: “I only trust my doctor, the one I started out with. So even if I were to move to another state, I have already asked would it be possible for me to just come back and see her because I know I wouldn’t want to see no other doctor.” (Male, 26 years old)

### Other healthcare workers

A number of participants also emphasized the important role of other healthcare workers in providing care. In general, participants highly valued an integrated approach to care, whereby they could receive support for other health-related matters, not just those directly related to HIV appointments and medication. An integrated approach could be achieved if those providing care to the patient—HIV clinicians, psychologists, physician’s assistants, social workers, peer navigators, and so forth—worked together as a team. Contrasting his current team of healthcare providers with his prior experiences, one 45-year old male participant reflected: “There’s more of, ‘Okay, you need this? I’m right on it.’… So that’s helpful.” On the contrary, not getting support in accessing other (social) services made another participant feel as though the healthcare providers were not being receptive to his needs.

On a related note, patients saw it as useful when they could meet not only the primary HIV clinician, but also others (e.g., a physician’s assistant, social worker, or some other professional on the care team) who would work together with the HIV clinician to give the patient more comprehensive support (providing answers to questions, finding resources, etc.). Working together with a team of providers was useful because it was not always possible to reach the main HIV clinician:

“Everybody’s in one unit…and if you need something, you could pretty much go to…get it from either [doctor]. If I was out of a med and I just happened to be seeing my psych doc, I’d be like, ‘Can you give me this?’ Even though my psych doctor wasn’t the one who prescribed it, it’s still on my list…. So they’re pretty much working together.”

(Female, 46 years old)

## Discussion

Our findings revealed the influential nature of components of the health system on PLWHA’s engagement in HIV care. Main components included aspects related to the healthcare workers themselves—their style of communication, availability, disposition, and ability to work together as a team—as well as more logistical matters related to appointment scheduling and infrastructure for appointment reminders and prescription refills.

Our findings support the idea that healthcare worker characteristics and features of the patient-provider relationship are influential for patients’ appointment attendance and medication adherence. On the subject of taking medications, Ammassari et al.’s systematic review of quantitative studies on barriers to ART adherence found mixed results as to whether satisfaction with the patient-provider relationship and healthcare received was associated with adherence [14]. However, that review along with a 2006 systematic review both indicated that the complexity/simplicity of the treatment and understanding the need for treatment were factors more consistently associated with ART adherence [14, 21]. Our findings demonstrate that perceptions about the treatment’s complexity/simplicity and about the necessity of treatment are related to the existence of appropriate patient-provider communication channels and the amount of information imparted by healthcare providers to patients about their treatment. Our data suggest further that adequate communication can help patients feel they are respected, regarded and known as persons by their providers. Another 2006 study, using cross-sectional analysis on a sample of 1,743 patients with HIV, found that there were statistically significant associations between patient perception of being “known as a person” by their provider and the outcomes of receiving ART, adhering to ART, and having undetectable serum HIV RNA [22].

With respect to appointment attendance, Flickinger et al.’s Baltimore-based observational study on whether provider communication and relationship factors influenced attending appointments found that “being known as persons by their providers” was the only factor significantly associated with attending appointments, after adjusting for demographic factors and substance abuse [18]. Our results suggest that a wider range of communication and relationship factors are also at play. Even amongst our relatively small group of study participants, we found that different ‘types’ of patient-provider relationships might be preferred, with some participants appreciating friendship in their clinicians, and others preferring a more professional relationship. Similarly, some participants looked forward to receiving as much information as possible, while others did not want providers to belabor things and ‘waste’ their time.

In light of the above, the takeaway might be that a flexible approach is best, where providers evaluate the kind of relationship most appropriate for a given patient or circumstance. Nevertheless, one general finding was that more holistic and readily available support, which could be provided by a team of medical personnel, seemed to be appreciated by all. In this regard, one of the few, in-depth qualitative studies on facilitators and barriers to HIV care among an urban, US-based population of PLWHA found that positive interactions with healthcare workers could increase motivation to engage in HIV care and availability of care could increase access to accurate information about HIV, both of which contribute to developing behavioral skills for “self-sustained retention” in care [23].

On the topic of appointment logistics, Smith et al. found that competing priorities leading to conflicts with appointment times were a barrier to retention in HIV care among a population of generally low-income, inner-city PLWHA based in the Bronx, NY [23]. Interestingly, the competing priorities—cited as family commitments, leisure activities, and treatment for substance abuse and other co-morbidities—were not reported by that population to have diminished the importance of attending HIV care appointments. In our study representing a very low-income population, conflicts with work obligations seemed to pose the most significant challenge, in fact obliging participants to prioritize getting a paycheck over visiting the doctor. The greater emphasis placed on competing work obligations by our sample compared to that of Smith et al.’s sample might be due to the fact that four-fifths of their participants were on disability or sick leave [23].

Among our participants, receiving reminders was cited as one of the most helpful facilitators for appointment attendance. This supports Smith et al.’s finding that strategies like using daily planners, calendars, and visible notes facilitated retention in care [23], as well as Park et al.’s finding that forgetting or confusing appointment times was one of the most common reasons for missing HIV clinic visits among a sample of urban-based PLWHA in South Korea [24]. We found that whether a text message, phone call, or letter was best depended on individual preference. As some participants lived with family members who did not know about their HIV status, privacy considerations should factor into the type of reminder used. Notably, beyond the ‘merely’ instrumental function of the reminder, Smith and colleagues reported that appointment reminder calls, among other health systems-level actions, made patients perceive the provider as being “personally responsive to their needs” and facilitated positive attitudes toward HIV care [23]. We have likewise observed that closer communication between the healthcare provider and the patient could have a motivating effect on engagement in care. The use of text messages could be an easy, low-cost and acceptable method for strengthening communication.

Long waiting times after arriving for appointments were a source of criticism by some, but not all, participants in our study. Excessive waiting could be seen as a reflection of not being valued or respected as a person, and therefore could contribute to a negative view of the health system. Alternatively, long waiting periods could be interpreted as a consequence of health worker shortage. However, it was not clear from our data whether either, if any, of these interpretations actually resulted in patients skipping an appointment. In the HIV literature, the impact of long waiting times on retention in care has been less examined. Clinic wait times were mentioned briefly as a structural barrier by Smith et al. [23], while in the non-HIV context there is some evidence that long patient wait times could affect likelihood of visiting the same healthcare provider again [25].

Our study also revealed some new areas for further investigation. Some of our participants’ statements about excessively frequent or long appointments hinted that they distrusted the healthcare system and providers, perceiving either ulterior motives (such as a desire to make money off of the patient’s visits) or incompetence (since so many/such long appointments were not perceived to be medically necessary, from the patient’s perspective). Thus, further research elucidating how to build trust and address perceptions that appointments are unnecessary could be useful.

Additionally, our results support the need for developing and testing interventions that combine comprehensive HIV care with support for logistical aspects related to appointment attendance and medication refills. Brennan et al.’s systematic review found relatively few RCTs testing interventions to link patients to, or motivate their retention in, HIV care [26]. However, the handful of studies that they did find revealed that interventions based on supporting patient self-management (e.g., through case management) could improve starting, and possibly staying in, HIV care. However, results on the effectiveness of delivery of services in a particular way were inconclusive. Only one study was reviewed, which tested the use of reminders, but the study was insufficiently powered to look for statistically significant differences in the sub-group of PLWHA [26, 27].

The major strength of this research lies in our use of in-depth qualitative methods to explore a significant public health challenge: engaging marginalized persons in HIV care. In our study, we targeted one of the highest-risk groups for non-retention in HIV care: low-SES, African American PLWHA, many of whom have had a history of substance abuse [28]. We listened to participants describe in their own words the challenges they faced in attending appointments and adhering to ART. We did not restrict them to pre-defined categories of barriers and facilitators, and the challenges pertaining to health systems described above emerged as some of the primary influencing factors. (An analysis of psychosocial factors is the topic of a separate forthcoming paper.)

Several challenges and limitations were faced in carrying out the mPeer2Peer study, which consequently affected our qualitative investigation. Because of the marginalized nature of the population, recruitment was a challenge and initially very slow. Thus, some of the initial inclusion criteria were revised to ensure sufficient participants (such as eliminating the requirement that the participant not currently be on ART, and revising downward the minimum time elapsed since seeing an HIV care provider from 12 months to 6 months). This may mean that our qualitative results did not capture those individuals in the most extreme situations of vulnerability, or that our results may not be transferable to that population. Also, to save participants from having to make an extra trip to the study center, some interviews took place immediately after the baseline assessment was conducted, which meant that the visit was long and participants might have been tired or eager to leave.

## Conclusions

Our study demonstrates the still-unmet need for providing comprehensive and effective HIV care to marginalized individuals. The multiple barriers cited by participants reflect the numerous points in the continuum of HIV care where a patient may fall through—from simply not being reminded about an appointment to feeling disrespected by healthcare providers; from not knowing what to do when an ART dose is skipped, to not having access to refills because the pharmacy is not open after school/work hours. At the same time, the various facilitators cited by study participants indicate the numerous opportunities that exist for improving quality of HIV care and means of delivering it. Among the facilitators mentioned, we found receiving appointment reminders, a good patient/provider-team relationship, and effective communication by clinicians to be particularly salient. These factors may represent important targets for future health systems-level interventions, which if successful, could make HIV care more patient-centered and accessible for the most vulnerable groups.

## References

[1] Detailed Tables for the National Vital Statistics Report (NVSR) “Deaths: Final Data for 2013”. Atlanta (GA): Centers for Disease Control and Prevention. 2013. Available from: http://www.cdc.gov/nchs/data_access/Vitalstatsonline.htm.

[2] Baltimore City Health Disparities Report Card 2013. Baltimore (MD): Baltimore City Health Department. 2014. Available from: http://health.baltimorecity.gov/sites/default/files/Health%20Disparities%20Report%20Card%20FINAL%2024-Apr-14.pdf.

[3] García de Olalla P, Knobel H, Carmona A, Guelar A, López-Colomés JL, JA C. Impact of adherence and highly active antiretroviral therapy on survival in HIV-infected patients. J Acquir Immune Defic Syndr. 2002 5;30(1):105–10. 12048370 10.1097/00042560-200205010-00014

[4] Lima VD, Harrigan R, Bangsberg DR, Hogg RS, Gross R, Yip B et al The combined effect of modern highly active antiretroviral therapy regimens and adherence on mortality over time. J Acquir Immune Defic Syndr. 2009 4;50(5):529–36. 10.1097/QAI.0b013e31819675e9 19223785 PMC3606956

[5] Martin M, Del Cacho E, Codina C, Tuset M, De Lazzari E, Mallolas J et al Relationship between adherence level, type of the antiretroviral regimen, and plasma HIV type 1 RNA viral load: a prospective cohort study. AIDS Res Hum Retroviruses. 2008 10;24(10):1263–68. 10.1089/aid.2008.0141 18834323

[6] Nachega JB, Hislop M, Dowdy DW, Chaisson RE, Regensberg L, M G. Adherence to nonnucleoside reverse transcriptase inhibitor-based HIV therapy and virologic outcomes. Ann Intern Med. 2007 4;146(8):564–73. 17438315 10.7326/0003-4819-146-8-200704170-00007

[7] Eldred L, F M. Introduction [to supplemental issue on the Health Resources and Services Administration Special Projects of National Significance Outreach Initiative]. AIDS Patient Care STDS. 2007;21(Suppl 1):S1–S2.

[8] Gardner EM, McLees MP, Steiner JF, Del Rio C, WJ B. The spectrum of engagement in HIV care and its relevance to test-and-treat strategies for prevention of HIV infection. Clin Infect Dis. 2011 3;52(6):793–800. 10.1093/cid/ciq243 21367734 PMC3106261

[9] Mugavero MJ, Norton WE, S MS. Health care system and policy factors influencing engagement in HIV medical care: piecing together the fragments of a fractured health care delivery system. Clin Infect Dis. 2011 1;52(Suppl 2):S238–46. 10.1093/cid/ciq048 21342913 PMC3106258

[10] Philbin MM, Tanner AE, DuVal A, Ellen J, Kapogiannis B, JD F. Linking HIV-positive adolescents to care in 15 different clinics across the United States: creating solutions to address structural barriers for linkage to care. AIDS Care. 2014 1;26(1) 10.1080/09540121.2013.808730 PMC3872213 23777542

[11] Rumptz MH, Tobias C, Rajabiun S, Bradford J, Cabral H, Young R et al Factors associated with engaging socially marginalized HIV-positive persons in primary care. AIDS Patient Care STDS. 2007;21(Suppl 1):S30–39. 17563288 10.1089/apc.2007.9989

[12] Yehia BR, Stewart L, Momplaisir F, Mody A, Holtzman CW, Jacobs LM et al Barriers and facilitators to patient retention in HIV care. BMC Infect Dis. 2015 6;15:246 10.1186/s12879-015-0990-0 26123158 PMC4485864

[13] Mugavero MJ, Amico KR, Horn T, MA T. The state of engagement in HIV care in the United States: from cascade to continuum to control. Clin Infect Dis. 2013 10;57(8):1164–71. 10.1093/cid/cit420 23797289

[14] Ammassari A, Trotta MP, Murri R, Castelli F, Narciso P, Noto P et al Correlates and predictors of adherence to highly active antiretroviral therapy: overview of published literature. J Acquir Immune Defic Syndr. 2002 12;31(Suppl 3):S123–27. 12562034 10.1097/00126334-200212153-00007

[15] Eberhart MG, Yehia BR, Hillier A, Voytek CD, Fiore DJ, Blank M et al Individual and community factors associated with geographic clusters of poor HIV care retention and poor viral suppression. J Acquir Immune Defic Syndr. 2015 5;69(Suppl 1):S37–43. 10.1097/QAI.0000000000000587 25867777 PMC4568746

[16] Giordano TP, Visnegarwala F, White AC Jr., Troisi CL, Frankowski RF, Hartman CM et al Patients referred to an urban HIV clinic frequently fail to establish care: factors predicting failure. AIDS Care. 2005 8;17(6):773–83. 16036264 10.1080/09540120412331336652

[17] Giordano TP, Hartman C, Gifford AL, Backus LI, RO M. Predictors of retention in HIV care among a national cohort of US veterans. HIV Clin Trials. 2009 Sep-Oct;10(5):299–305. 10.1310/hct1005-299 19906622

[18] Flickinger TE, Saha S, Moore RD, MC B. Higher quality communication and relationships are associated with improved patient engagement in HIV care. J Acquir Immune Defic Syndr. 2013 7;63(3):362–66. 10.1097/QAI.0b013e318295b86a 23591637 PMC3752691

[19] Vlahov D, Anthony JC, Munoz A, Margolick J, Nelson KE, Celentano DD et al The ALIVE study, a longitudinal study of HIV-1 infection in intravenous drug users: description of methods and characteristics of participants. National Institute on Drug Abuse Research Monograph Series. 1991;109:75–100. 1661376

[20] Braun V, V C. Using thematic analysis in psychology. Qual Res Psychol. 2006 7;3:77–101.

[21] Mills EJ, Nachega JB, Bangsberg DR, Singh S, Rachlis B, Wu P et al Adherence to HAART: a systematic review of developed and developing nation patient-reported barriers and facilitators. PLoS Medicine. 2006 11;3(11):2039–64. 10.1371/journal.pmed.0030438 PMC1637123 17121449

[22] Beach MC, Keruly J, RD M. Is the quality of the patient-provider relationship associated with better adherence and health outcomes for patients with HIV? J Gen Intern Med. 2006 6;21(6):661–65. 16808754 10.1111/j.1525-1497.2006.00399.x PMC1924639

[23] Smith LR, Fisher JD, Cunningham CO, KR A. Understanding the behavioral determinants of retention in HIV care: a qualitative evaluation of a situated information, motivation, behavioral skills model of care initiation and maintenance. AIDS Patient Care STDS. 2012 6;26(6):344–55. 10.1089/apc.2011.0388 22612447 PMC3366334

[24] Park WB, Kim JY, Kam SH, Kim HB, Kim NJ, O MD et al Self-reported reasons among HIV-infected patients for missing clinic appointments. Int J STD AIDS. 2008 2;19(2):125–26. 10.1258/ijsa.2007.007101 18334069

[25] Hill CJ, J K. The impact of unacceptable wait time on health care patients’ attitudes and actions. Health Mark Q. 2005;23(2):69–87. 17182462 10.1300/J026v23n02_05

[26] Brennan A, Browne JP, H M. A systematic review of health service interventions to improve linkage with or retention in HIV care. AIDS Care. 2014;26(7):804–12. 10.1080/09540121.2013.869536 24354712

[27] Perron NJ, Dao MD, Kossovsky MP, Miserez V, Chuard C, Calmy A et al Reduction of missed appointments at an urban primary care clinic: a randomised controlled study. BMC Fam Pract. 2010 10;11:79 10.1186/1471-2296-11-79 20973950 PMC2984453

[28] Horstmann E, Brown J, Islam F, Buck J, BD A. Retaining HIV-infected patients in care: Where are we? Where do we go from here? Clin Infect Dis. 2010 2;50(5):752–61. 10.1086/649933 20121413

